# Tumor microenvironment characterization in colorectal cancer to identify prognostic and immunotherapy genes signature

**DOI:** 10.1186/s12885-023-11277-4

**Published:** 2023-08-18

**Authors:** Xian-wen Guo, Rong-e Lei, Qing-nan Zhou, Guo Zhang, Bang-li Hu, Yun-xiao Liang

**Affiliations:** 1 Department of Gastroenterology, The People’s Hospital of Guangxi Zhuang Autonomous Region, No.6 Tao-Yuan Road, Nanning, 530021 Guangxi China; 2 Department of Gastroenterology, First Affiliated Hospital of Guangxi Medical University, Nanning, 530021 Guangxi China; 3 Department of Research, Guangxi Medical University Cancer Hospital, No.71 Hedi Road, Nanning, 530021 Guangxi China

**Keywords:** Tumor microenvironment, Colorectal cancer, Prognosis, Immunotherapy

## Abstract

**Background:**

The tumor microenvironment (TME) plays a crucial role in tumorigenesis, progression, and therapeutic response in many cancers. This study aimed to comprehensively investigate the role of TME in colorectal cancer (CRC) by generating a TMEscore based on gene expression.

**Methods:**

The TME patterns of CRC datasets were investigated, and the TMEscores were calculated. An unsupervised clustering method was used to divide samples into clusters. The associations between TMEscores and clinical features, prognosis, immune score, gene mutations, and immune checkpoint inhibitors were analyzed. A TME signature was constructed using the TMEscore-related genes. The results were validated using external and clinical cohorts.

**Results:**

The TME pattern landscape was for CRC was examined using 960 samples, and then the TMEscore pattern of CRC datasets was evaluated. Two TMEscore clusters were identified, and the high TMEscore cluster was associated with early-stage CRC and better prognosis in patients with CRC when compared with the low TMEscore clusters. The high TMEscore cluster indicated elevated tumor cell scores and tumor gene mutation burden, and decreased tumor purity, when compared with the low TMEscore cluster. Patients with high TMEscore were more likely to respond to immune checkpoint therapy than those with low TMEscore. A TME signature was constructed using the TMEscore-related genes superimposing the results of two machine learning methods (LASSO and XGBoost algorithms), and a TMEscore-related four-gene signature was established, which had a high predictive value for discriminating patients from different TMEscore clusters. The prognostic value of the TMEscore was validated in two independent cohorts, and the expression of TME signature genes was verified in four external cohorts and clinical samples.

**Conclusion:**

Our study provides a comprehensive description of TME characteristics in CRC and demonstrates that the TMEscore is a reliable prognostic biomarker and predictive indicator for patients with CRC undergoing immunotherapy.

## Background

Colorectal cancer (CRC) is the third most common cancer and the fourth leading cause of cancer-related deaths, according to a recent report by GLOBOCAN 2020 [1]. Numerous advancements have been made over the past few decades with regard to CRC treatment, and the five-year survival rate has increased significantly [2]. Recently, immunotherapy, such as immune-checkpoint blockade programmed cell death 1 (PD-1) and programmed cell death 1 ligand 1 (PD-L1), has further improved the prognosis of patients with CRC, especially those with late-stage cancer [3, 4]. However, owing to the heterogeneity of CRC, only a subset of patients is suitable for immunotherapy [5, 6]; therefore, it is essential to investigate the pathogenesis of CRC and screen patients that may be appropriate for immunotherapy.

Tumor components are very complex; besides tumor cells, there are numerous immune, stromal, and inflammatory cells in the tumor microenvironment (TME) that modulate tumor development and other biological functions [7]. Immune cells in the TME are associated with the pathogenesis, development, therapeutic response, and prognosis of various cancers. Immune cell abundance varies greatly in tumor tissues, and different types of immune cells display distinct functions that exert pro- and anti-tumor effects at different stages [8]. A dynamic balance exists between the pro- and anti-tumor effects within the TME, profoundly influencing the prognosis of patients with cancer [9].

Recently, the abundance of immune and other cells in the TME has been assessed quantitatively using computational methods. This has been termed the TMEscore, which has great value in identification of patients suitable for precision therapy or immunotherapy in several cancers, such as gastric [10], bladder [11] and prostate cancer [12]. However, the comprehensive landscape of the TME cells in CRC has not yet been characterized, and the utility of the TMEscore for CRC remains to be elucidated. Therefore, the aim of the present study was to estimate the TME patterns of CRC using a meta-cohort with larger tumor samples. Additionally, we intended to systematically analyze the TMEscore with genomic characteristics and CRC clinical out, which could provide key biomarkers for predicting responsiveness to immunotherapy, thereby further improving precision immunotherapy for CRC.

## Materials and methods

### CRC dataset acquisition and preprocessing

CRC datasets were systematically searched from the Gene Expression Omnibus (GEO) database (https://www.ncbi.nlm.nih.gov/geo/), and only datasets with sufficient clinical information were collected. In total, five datasets were selected (GSE103479, GSE29621, GSE72970, GSE39582, and GSE41258) with survival information regarding samples from patients with CRC. Four datasets (GSE20916, GSE21815, GSE3629, and GSE89287) with tumor tissues and the corresponding control tissues were also downloaded. We downloaded The Cancer Genome Atlas (TCGA)-COADREAD dataset (615 samples) from the TCGA database (GDC hub: https://gdc.xenahubs.net), which included 449 colon cancer and 166 rectal cancer samples, and we also downloaded the corresponding clinical information. The raw data from the GEO datasets were preprocessed using the RMA algorithm for background adjustment before analysis. The raw data from the TCGA dataset were transformed into transcripts per kilobase million values prior to analysis. The “sva” R package was used to process the RNA expression data from both the GEO and TCGA cohorts with the aim of reducing the batch effect.

### Quantification of tumor infiltrating cells, immune scores, and tumor purity

The CIBERSORT algorithm [13] was used to quantify the proportions of immune infiltrating cells in CRC samples, which allowed for sensitive and specific discrimination of 22 human immune cell phenotypes by calculating gene expression. The immune score, stromal score, Estimation of Stromal and Immune cells in Malignant Tumor tissues using Expression data (ESTIMATE) score, and tumor purity of CRC samples were calculated using the ESTIMATE algorithm [14]. This algorithm provides researchers with scores for tumor purity, the level of stromal cells present, and the infiltration level of immune cells in tumor tissues based on gene expression.

### Consensus clustering analysis

Unsupervised clustering methods for dataset analysis (TME pattern and TMEscore) were used to classify samples and determine the optimal number of clusters for further analysis. The procedures were undertaken using the ConsensusClusterPlus package [15] in R software, and were repeated 1,000 times, to ensure classification stability. The tool implements the consensus clustering method and provides visualizations, including item tracking, item-consensus, and cluster-consensus plots. The results are in the form of cumulative distribution function curves, which are used to determine the number of clusters, with k values ranging from 2 to 6. Furthermore, a consensus heatmap of the clusters was determined and visualized.

### Differentially expressed gene analysis

For the GEO datasets, the differentially expressed genes (DEGs) among different groups were screened using the “limma” package, which adopts an empirical Bayesian approach to estimate gene-expression changes using moderated *t* tests. For the TCGA dataset, DEGs were screened using the “edgeR” package, which is specifically used to screen DEGs in TCGA datasets. The DEGs were determined based on the |logFC| > 0.5 and *P* value < 0.01 criteria.

### Functional and pathway enrichment analysis

Gene annotation enrichment analysis was performed on DEGs from TMEscore-related genes using the clusterProfiler R package [16]. Gene Ontology (GO) and Kyoto Encyclopedia of Genes and Genomes (KEGG) terms of the genes were identified with a cutoff of *P* < 0.01 and a false discovery rate < 0.05. Gene set variation analysis (GSVA) was used to screen significantly enriched pathways between the two clusters using the Molecular Signatures Database (MSigDB) version 7.4 [17]. Significant pathway terms were set at a *P*-value < 0.05.

### TMEscore calculation and establishment of the TME signature

The “TMEscore” package [10, 18] was used to calculate the TMEscore and establish TME clusters for the gene datasets, which estimates the TMEscore based on gene expression. This provides functionality for calculating the TMEscore using principal component analysis or z-score methods. The results generated four types of data: TMEscoreA, TMEscoreB, TMEscore, and TME_binary. TME_binary divides the samples into high or low clusters based on the TMEscore value.

### Reverse transcription-polymerase chain reaction (RT-PCR) assay using CRC clinical samples

Gene expression in clinical CRC samples was tested using RT-PCR. Forty tumor tissues and their corresponding adjacent tissues were collected from our hospital between February 2020 and August 2021. Total RNA from the tissues was isolated using TRIzol reagent (Invitrogen, Waltham, MA, USA), according to the manufacturer’s instructions. The primers used for RT-PCR are as follows: CXCL10 FORWARD: CTC TCT CTA GAA CTG TAC GCT G, REVERSE: ATT CAG ACA TCT CTT CTC ACC C; IDO1 FORWARD: CTG CCT GAT CTC ATA GAG TCT G, REVERSE: TTG TGG TCT GTG AGA TGA TCA A; MAB21L2 FORWARD: CTA TCT CTC AGC GCG TAA GAT C, REVERSE: CAT CTT GAC CAC ATC CCG ATA G; LZTS2 FORWARD: TCC TCC TCC TCC TCT TCC TCC TC, REVERS: GCA GGC TGG ACA GTG AGT TTCG. RT-PCR was performed using the SYBR ® Premix Ex Taq kit (Takara, Dalian, China). The relative expression of each gene was calculated using the 2^−ΔΔCT^ method. This study was approved by the hospital’s ethics committee.

### Statistical analysis

Statistical significance for normally distributed variables was tested using the unpaired Student’s *t* test when comparing two groups; otherwise the Mann–Whitney U test was used. The Kruskal–Wallis test and one-way Analysis of Variance were used as nonparametric and parametric methods, respectively, for the comparison of more than two groups. The Chi-square test was used to compare categorical variables. Kaplan–Meier and log-rank (Mantel–Cox) tests were used to determine the statistical significance of patient survival. Two machine learning methods, LASSO regression and XGBoost, were used to screen significant genes using the “glmnet” and “xgboost” packages, respectively. Receiver Operating Characteristic (ROC) curves were used to calculate the area under the curve (AUC) and evaluate the predicted value of the signature. All statistical analyses were performed using R language. *P-*values were two-sided, and P < 0.05 were considered to indicate statistical significance.

## Results

### Construction and analysis of TME landscape patterns in CRC

**Fig. 1:**
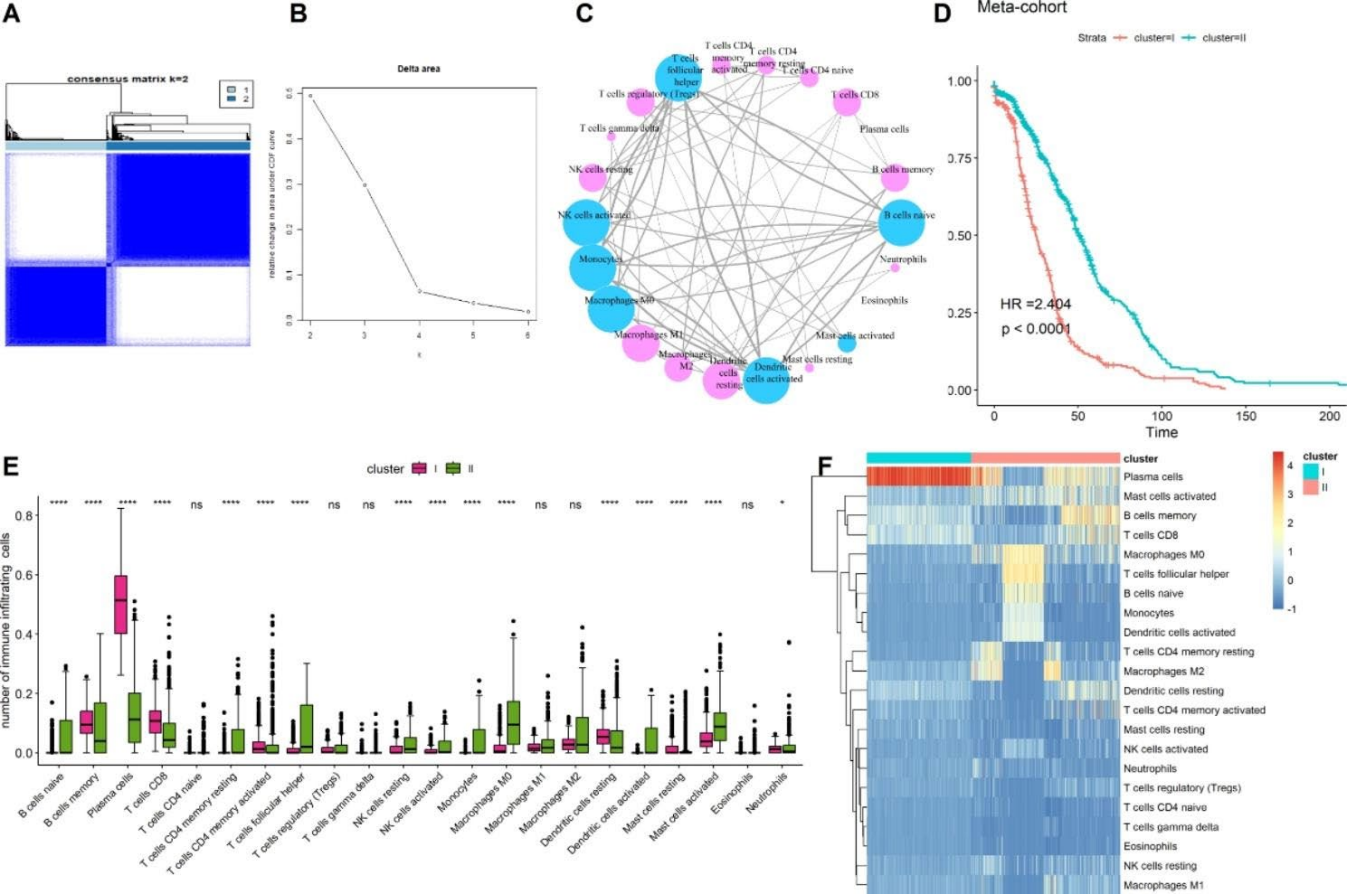
Landscape of TME patterns in four CRC cohorts (TCGA-CORDREAD, GSE103479, GSE29621 and GSE72970) with 960 samples. (**A**) Heatmap of the consensus clustering matrix for k = 2; (**B**) CDF curves of the consensus score from k = 2 to 6. (**C**) Cellular interaction of the TME cell types. Cell cluster I: blue; cell cluster II: pink. The size of each cell represents survival of each TME cells type, the smaller log-rank test P value, the larger size of node. The lines connecting TME cells represent cellular interactions. The thickness of the line represents the strength of correlation estimated by Spearman correlation analysis. The more correlation, the more thickness of the lines. (**D**) Kaplan–Meier curves for overall survival (OS) of 960 CRC patients from four CRC cohorts between two TME clusters. (**E**) Comparison of 22 immune cells infiltrating fraction between TME cluster I and cluster II. (**F**) Unsupervised clustering of 22 immune cells in TME for 960 patients from four CRC cohorts

The TME pattern of CRC was evaluated by calculating the immune cell infiltrating fraction from four CRC datasets (TCGA-CORDREAD, GSE103479, GSE29621, and GSE72970) with a total of 960 CRC tissue samples, using the CIBERSORT algorithm. After removing the batch effect for the datasets, each immune cell infiltrating fraction was combined into a large meta-cohort to construct the landscape of the TME pattern. Subsequently, an unsupervised hierarchical clustering method using the ConsensusClusterPlus package was used to determine the optimal cluster number for the TME pattern, and two robust clusters (clusters I and II) of the meta-cohort were identified (Fig. 1A and B). Next, the correlation of each type of immune cell was analyzed, and the TME cell network was visualized as a landscape of CRC tumor–immune cell interactions and CRC cell lineages (Fig. 1C and F). Based on the two clusters of TME cell patterns, we found that the patients with CRC in cluster II had better prognosis than those in cluster I (log-rank test, *P* = 0.001; HR = 2.404; Fig. 1D). We also found that the infiltrating fractions of plasma, T, CD8, and resting dendritic cells were considerably higher in cluster I than in cluster II. In addition, the infiltrating fraction of Macrophages M0, T cells, follicular helper T cells, and resting CD4 memory cells were significantly higher in cluster II than in cluster I (Fig. 1E).

### Identification of TME clusters in TCGA dataset

**Fig. 2:**
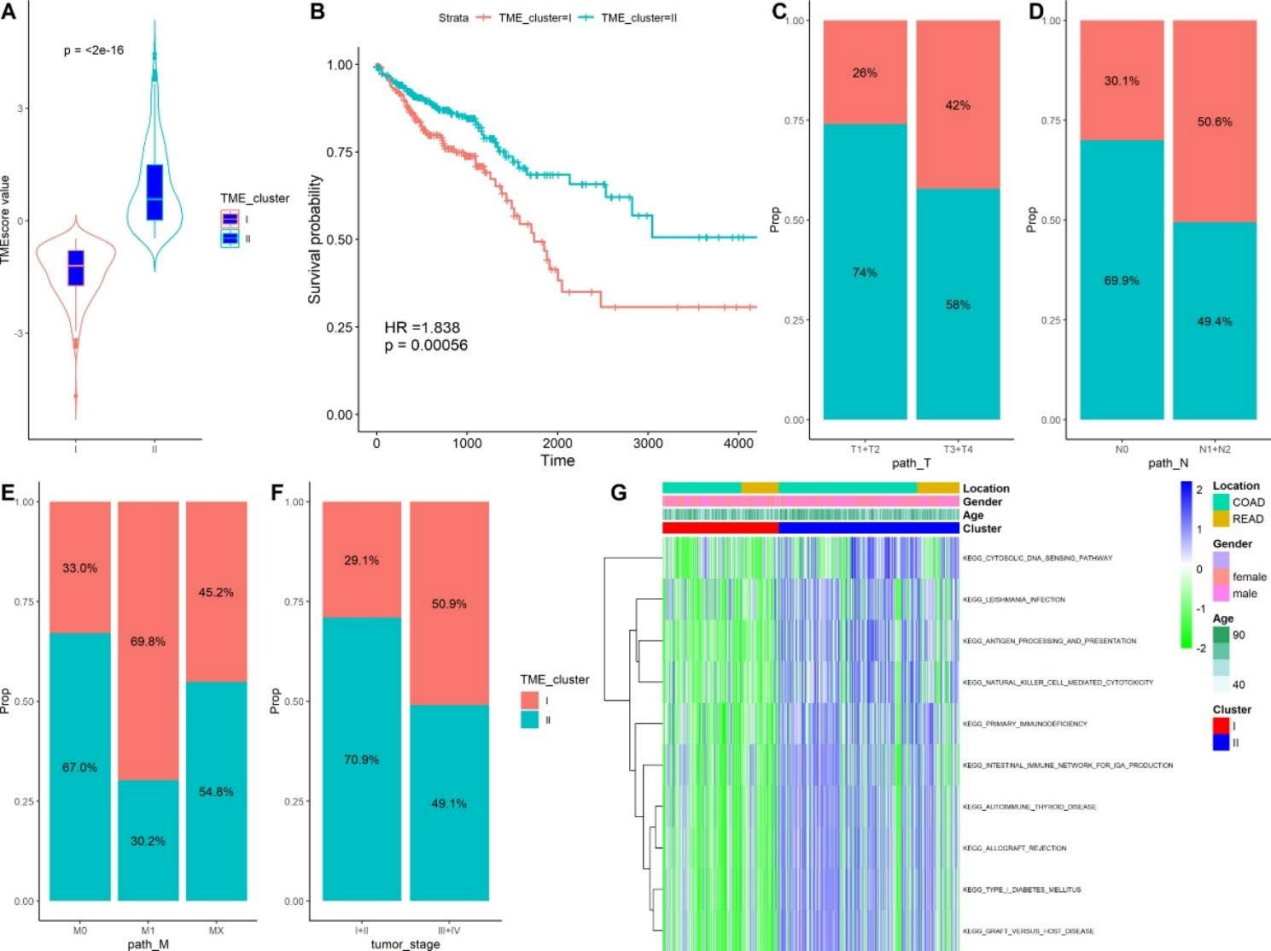
Identification of TME clusters in TCGA dataset. (**A**) Comparison of TME scores between TME-cluster I and TME-cluster II. (**B**) Kaplan–Meier curves for overall survival of CRC patients between two TME clusters. Comparison of T stage (**C**), N stage (**D**), M stage (**E**), Tumor stage (**F**) between TME-cluster I and TME-cluster II. (**G**) GSVA method revealed the pathways that the TME-clusters involved

To characterize the clinical significance of the TME pattern between the two clusters, we focused on the TCGA-CORDREAD dataset, which contains comprehensive clinical information for most patients. Here, we calculated the immune cell infiltrating fraction for the TCGA dataset, and the TMEscore of the immune cells was calculated using the “TMEscore” package. Subsequently, unsupervised hierarchical cluster analysis was performed to screen the optimal clusters for the TMEscore. Two distinct clusters were identified for the TMEscore (TME-cluster I and TME-cluster II). TME-cluster II was associated with a higher immune score than TME-cluster I (Kruskal–Wallis, *P* < 0.001; Fig. 2A). Survival analysis revealed that patients in TME-cluster II exhibited better survival rates than those in TME-cluster I (log-rank test, *P* = 0.001; HR = 1.838; Fig. 2B). The clinical characteristics analysis results indicated that patients in TME-cluster II exhibited increased MSI-H status, T1 + T2 stage, N0 stage, M0 stage, and I + II tumor stage than those in TME-cluster I (Fig. 2C–F); however, there was no significant difference in sex or tumor location. The results indicate that patients in TME-cluster II displayed high TMEscores and were associated with early-stage CRC. Subsequently, we explored the pathways involving the two TME clusters using the GSVA method, and observed immunodeficiency, antigen processing and presentation, and natural killer cell-mediated cytotoxicity pathways underlying the TME clusters (Fig. 2G).

### Construction of the TME signature and functional annotation

**Fig. 3:**
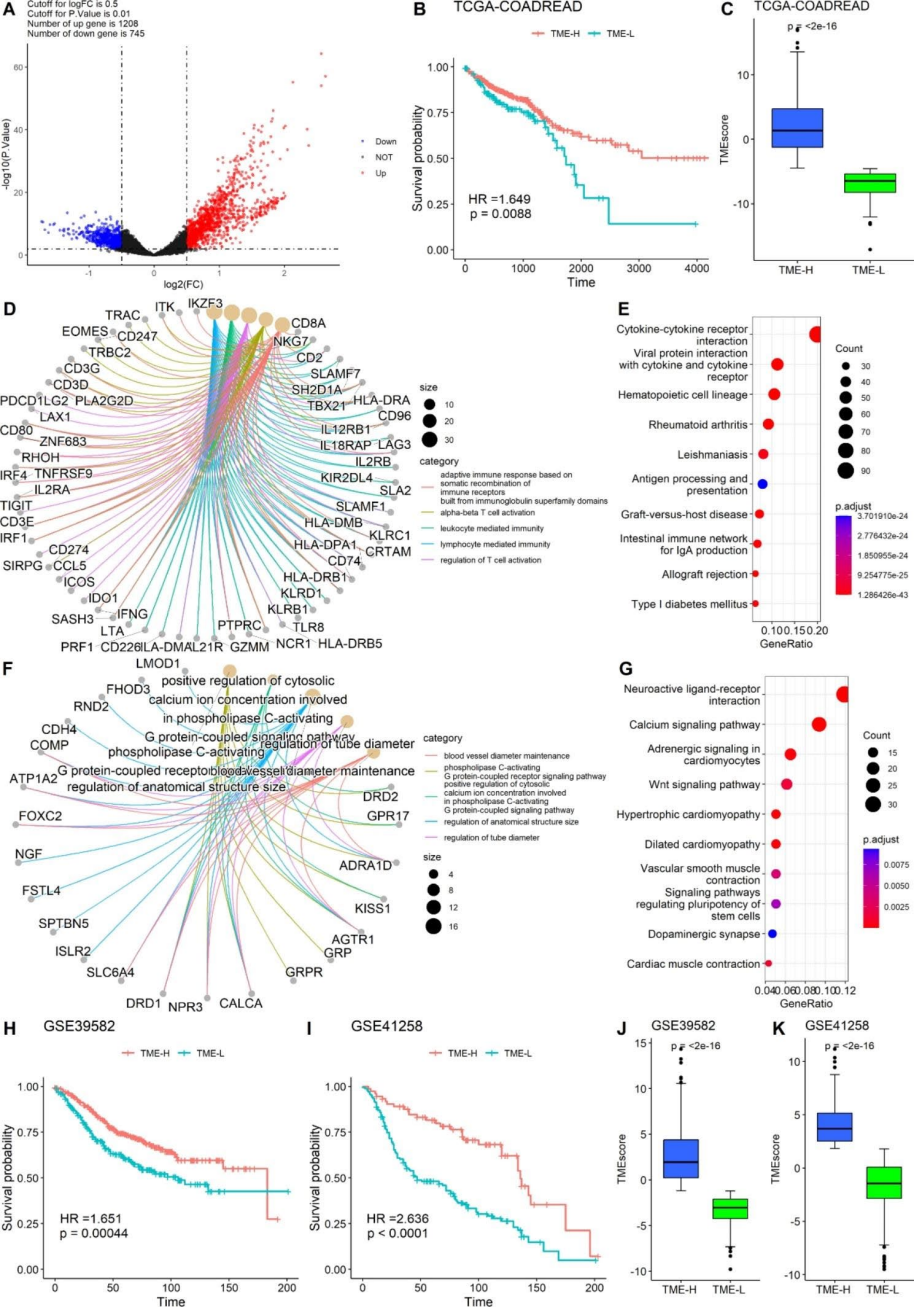
Construction of the TME signature and functional annotation. (**A**) Volcano plot for the differential expressed genes (DEGs) between TME-cluster I and TME-cluster II. (**B**) Kaplan–Meier curves for overall survival of CRC patients between TME-H and TME-L. (**C**) Comparison of immune scores between TME-H and TME-L signature. (**D-E**) GO and KEGG analysis for the upregulated genes. (**F-G**) GO and KEGG analysis for the downregulated genes. Validated survival impact of TME signature using the DEGs in (**H**) GSE39582 dataset and (**I**) GSE41258 dataset. Validated immune scores of TME signatures using the DEGs in (**J**) GSE39582 dataset and (**K**) GSE41258 dataset

To further determine the underlying biological characteristics of TME clusters, we screened DEGs between TME-cluster I and TME-cluster II of the TCGA dataset using the “edgeR” package. Here, 1953 DEGs were identified based on the (|logFC| > 0.5 and *P* value < 0.01) criteria, with 1208 upregulated and 745 downregulated genes (Fig. 3A). The DEGs were then incorporated into the unsupervised hierarchical cluster analysis, and two clusters, TME-H and TME-L, were identified. The samples in the two signatures were significantly consistent with the clustering results of TME-clusters I and II (χ^2^ tests, *P* < 0.05). Survival analysis showed that patients with TME-H signature displayed a better prognosis than those with TME-L signature (log-rank test, *P* = 0.001; Fig. 3B). The results also revealed that the TME-H signature exhibited an elevated immune score (Fig. 3C).

GO enrichment and KEGG analyses of the signature genes were conducted using the R package clusterProfiler. We observed that upregulated genes were involved in the biological processes of leukocyte-mediated immunity, T-cell activation, and lymphocyte-mediated immunity. The pathways are involved in cytokine–cytokine receptor interaction and viral protein interaction with cytokines and cytokine receptors. The downregulated genes were involved in the phospholipase C-activating G protein-coupled receptor signaling pathway and positive regulation of cytosolic calcium ion concentration involved in the phospholipase C-activating G protein-coupled signaling pathway. The calcium and Wnt signaling pathways were found to be involved (Fig. 3D–G). Subsequently, we tested the robustness of the TME signature using DEGs in the GSE39582 and GSE41258 datasets. The results verified that the TME signatures constructed by DEGs in the GSE39582 and GSE41258 datasets were consistent to the TCGA-COADREAD dataset, and patients with CRC with the TME-H signature displayed a better prognosis (Fig. 3H and I). Finally, the correlations between TME signature scores and TME patterns were determined, and the TME-H signature was associated with elevated immune scores in the TCGA-COADREAD, GSE39582, and GSE41258 datasets (Fig. 3J and K), suggesting the reliability of the TME signature.

### Association of TME signature with the immune environment and immune checkpoint inhibitors

**Fig. 4:**
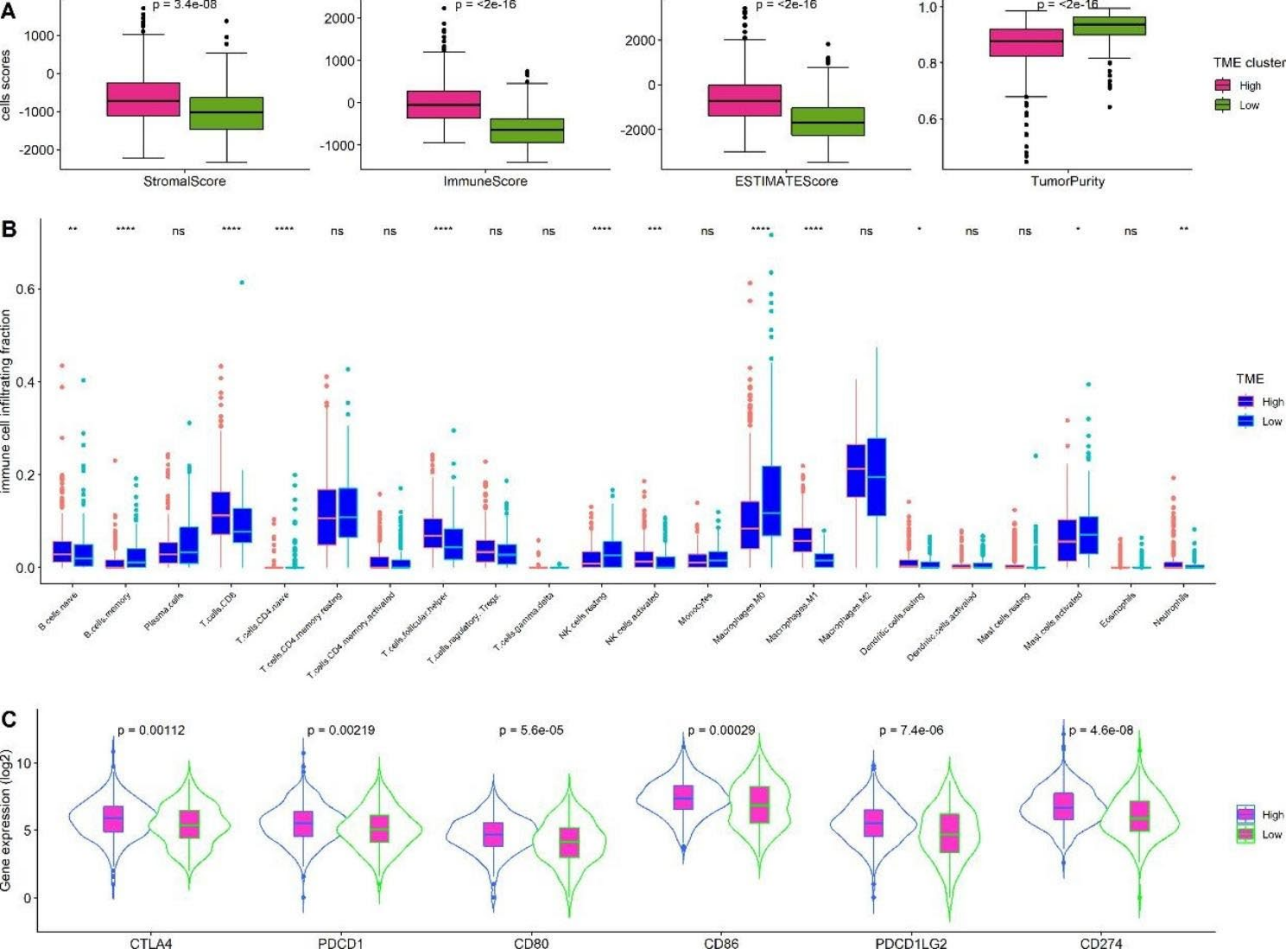
TME signature was associated with (**A**) immune cells scores and tumor purity; (**B**) tumor immune cells fraction; (**C**) immune checkpoint inhibitors

To determine the association of the TME signature with immune cell scores and tumor purity in CRC, we used the ESTIMATE algorithm to quantify the composition of CRC. The results indicated that TME-H had higher immune, stromal, and ESTIMATE scores, and lower tumor purity, than the TME-L signature (*P* < 0.05, Fig. 4A), demonstrating high abundances of immune and stromal cells and low tumor purity in patients with the TME-H signature. Next, we examined the association between the TME signature and immune cell infiltration fraction in CRC. As Fig. 4B illustrates, follicular helper T cells and M1 macrophages were more abundant in the TME-H cluster, whereas other immune cell infiltrating fractions showed little difference between the TME-H and TME-L signatures (*P* > 0.05). Finally, we examined the associations between the TME signatures with known immune checkpoint inhibitors, including CTLA4, PD1 (PDCD1), CD80, CD86, PD-L1, PD-L2 (PDCD1LG2), and (CD274). As shown in Fig. 4C, the expression levels of the six immune checkpoint inhibitors were increased in the TME-H when compared with those in the TME-L signature. The findings demonstrate that the TME signature is critically associated with the immune environment and may be used to screen patients with CRC who are suitable for immunotherapy.

### Association of TME signature with gene mutations and tumor mutation burden

**Fig. 5:**
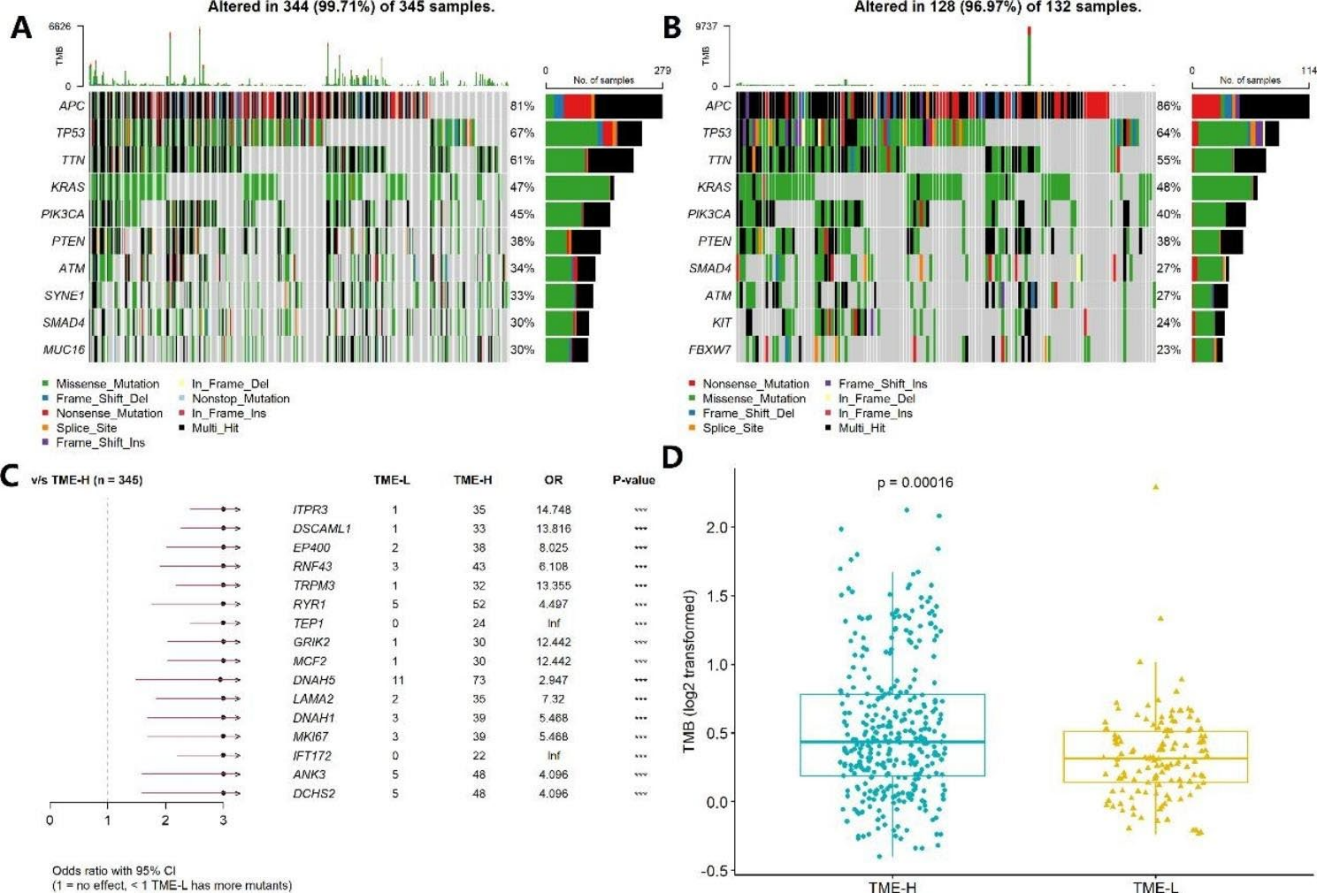
TME signature was associated with gene mutation and TMB. (**A-B**) Top 10 mutated genes in the TME-H and TME-L signature. (**C**) Comparison of gene mutation between TME-H and TME-L clusters. (**D**) Comparison of tumor mutation burden between TME-H and TME-L signature

Gene mutation and Tumor Mutation Burden (TMB) are closely related to the pathogenesis of various cancers, including CRC [19, 20]. Therefore, we explored the association of the TME signature with CRC gene mutations and TMB in the TCGA dataset using the “maftools” package. We first examined the whole gene mutation between the TME-H and TME-L signatures and found that the whole mutated frequency was higher in TME-H than in TME-L (99.71% vs. 96.97%). However, the top 10 mutated genes of the two signatures were similar, namely *APC*, *TP53*, *TTN*, *KRAS*, *PIK3CA*, *PTEN*, *ATM*, *SYNE1*, *MUC16*, and *SMAD4*, demonstrating that the two signatures harbor similar top mutated genes (Fig. 5A and B). Next, we screened the genes with significant differential mutation frequency between the two signatures and revealed that *ITPR3*, *DSCAML1*, *EP400*, *RNF43*, *TRPM3*, *RYR1*, *TEP1*, *GRIK2*, *MCF2*, and *DNAH5* showed higher mutation frequencies in the TME-H signature than in the TME-L signature. However, we did not observe a significant difference in the top 10 mutated genes between the two signatures (Fig. 5C). In addition, we observed that the TMB value was much higher in the TME-H signature than in the TME-L signature (*P* < 0.001; Fig. 5D). The findings indicate that the TME-H signature harbored higher gene mutations and TMB than the TME-L signature.

### Establishment of a predictive model for the TME signature

**Fig. 6:**
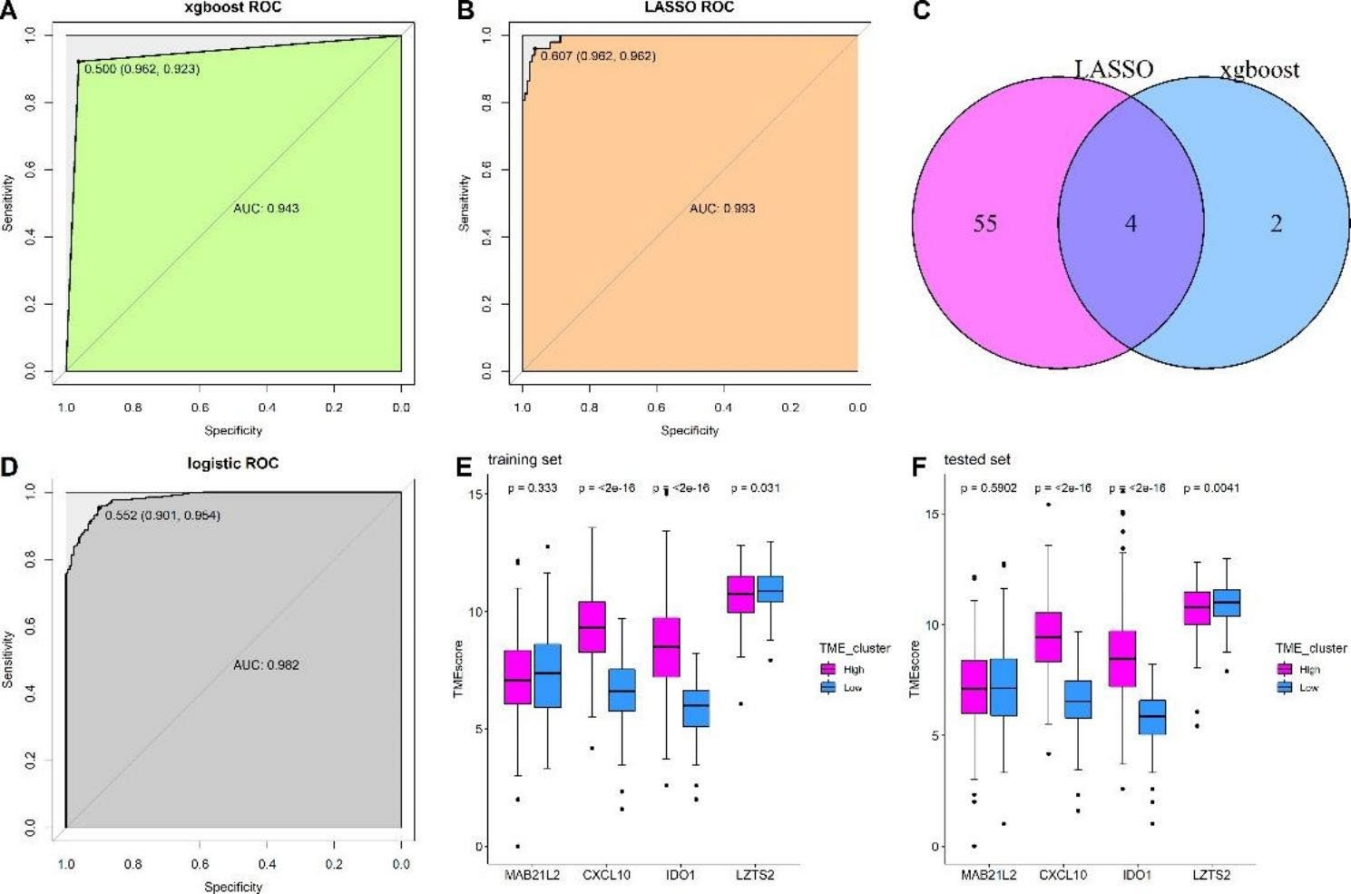
Establishment of predictive model for TME signature. (**A**) xgboost algorithms identified a model predicting the TME signature; (**B**) LASSO algorithms identified a model predicting the TME signature. (**C**) Venn diagram identified overlapped genes between xgboost and LASSO algorithms. (**D**) Multivariate logistic regression model revealed the predictive value for the TME signature. (**E**) Expression of the four overlap genes in training dataset between TME-H and TME-L signature. (**F**) Expression of the four overlap genes in tested dataset between TME-H and TME-L signature

To establish a predictive model for the TME signature, the CRC samples were randomly divided into two sets (training and test sets) at a 7:3 ratio as previous studies did [21, 22], with 430 and 185 samples in each set, respectively. Two machine learning algorithms (LASSO and XGBoost) were employed, by incorporating TMEscore DEGs into the model, and the key genes related to the TME signature were screened. Five-fold cross validation was performed to search the best hyper-parameters, and the “nround” was 25 in the present analysis, and the min logloss and min logloss index were 2.54 and 43, respectively. The LASSO and XGBoost algorithms identified 59 and 6 genes associated with the TME signature, respectively. The ROC curve showed that both models constructed using LASSO and XGBoost algorithms had good predictive value in differentiating TME-H from the TME-L signature (both AUC values > 0.90; Fig. 6A and B). We then superimposed the genes from the two algorithms and observed that four genes (*CXCL10*, *LZTS2*, *IDO1*, and *MAB21L2*) were identified by the two machine learning algorithms (Fig. 6C). Subsequently, we applied a multivariate logistic regression analysis to construct a predictive model using the four genes. As shown in Fig. 6D, the optimal cutoff value of the model for discriminating the two clusters was 0.552, which means that patients with scores < 0.552 were grouped in the TME-H signature; otherwise, they were grouped in the TME-L signature. The ROC curve demonstrated that the predictive value of the model reached 0.982, with a sensitivity and specificity of 90.1% and 95.4%, respectively, for the entire dataset. Finally, we tested the expression of the four genes in the training and test sets between the TME-H and TME-L signatures. The results showed that *CXCL10* and *IDO1* expression levels increased, while *LZTS2* levels decreased in the TME-H signature compared with that in TME-L signature; however, there was no significant difference between the two signatures regarding *MAB21L2* expression (Fig. 6E and F).

### Validation of TME signature-related genes in independent cohorts and CRC clinical samples

**Fig. 7:**
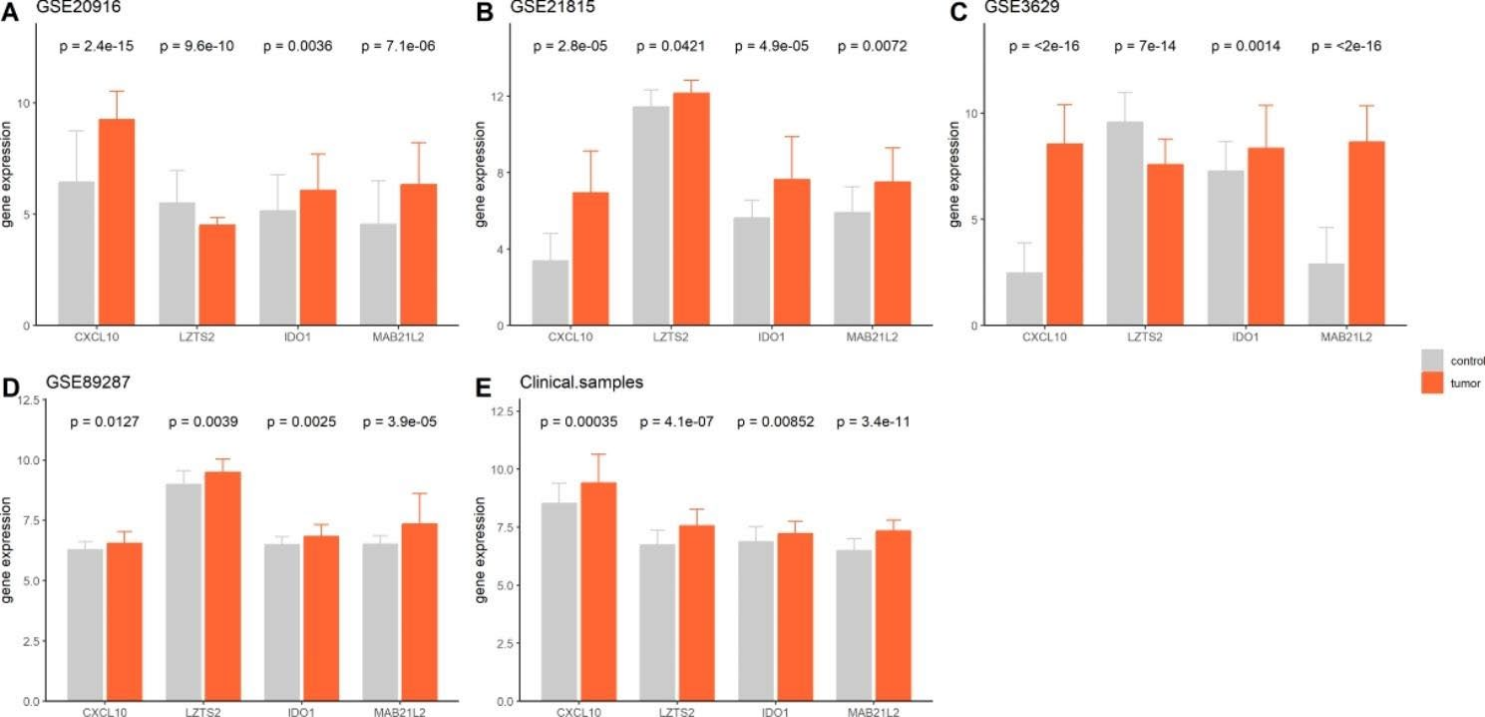
Validation of four genes in datasets and clinical samples. Comparison of CXCL10, LZTS2, IDO1 and MAB21L2 between CRC and control tissues in (**A**) GSE20916 dataset; (**B**) GSE21815 dataset; (**C**) GSE3629 dataset; (**D**) GSE89287 dataset; (**E**) clinical tissues

The expression of four TME-related genes (*CXCL10*, *LZTS2*, *IDO1*, and *MAB21L2*) that predict the TME signature of CRC was validated in four GEO datasets: GSE20916 (145 samples), GSE21815 (141 samples), GSE3629 (121 samples), and GSE89287 (71 samples), which contained normal control and tumor samples. Furthermore, 40 clinical CRC tissues and corresponding adjacent tumor tissues were collected, and the expression of the four genes was determined using RT-PCR assay. As shown in Fig. 7, the four GEO datasets and our clinical samples confirmed that the expression levels of *CXCL10*, *IDO1*, and *MAB21L2* were significantly higher in tumor tissues than in control tissues (*P* < 0.05). Moreover, the expression level of *LZTS2* decreased in tumor tissues from the GSE20916 and GSE3629 datasets, but appeared increased in samples from GSE21815 and GSE89287 datasets and our clinical samples (*P* < 0.05). Collectively, the results indicated that the levels of the three TME signature genes (*CXCL10*, *IDO1*, and *MAB21L2*) were all increased in CRC tissues when compared with the control tissues, but the expression of *LZTS2* in CRC tissues remained need to further validate.

## Discussion

Increasing evidence indicates that the TME profoundly influences tumorigenesis, progression, and therapeutic response; however, the utility of the TME in clinical settings is hampered by the inaccurate combination and uncertain interactions of the TME [23, 24]. Over the past few decades, flow cytometry or immunohistochemistry assays have mostly been used to determine various cell types in clinical tissues; however, these methods are subject to cumbersome procedures and low feedback efficiency, which limits the application of the TME in the clinical setting. With the development of sequencing techniques and bioinformatics, many deconvolution methods are available for predicting cell types and proportion information in various tissues [25, 26]. TMEscore, a parameter designed to quantitatively evaluate the composition of the TME using computational algorithms, was recently introduced as a biomarker for predicting the prognosis of patients with cancers and to guide more effective immunotherapy strategies. However, a comprehensive characterization of the TME in CRC has not yet been performed. The present study demonstrated that TMEscore and its signature are promising tools [10, 18].

Herein, we performed a systematic analysis to explore the value of the TMEscore with the clinical outcome in patients with CRC based on several gene datasets. We first depicted the TME cell landscape using a large meta-cohort, divided the cells into two clusters, and observed that the clusters were associated with the survival of patients with CRC. We then focused on the TCGA dataset and calculated its TMEscore, and the cluster analysis revealed its significant prognostic value and clinical significance, with a high TMEscore indicating better patient prognosis. Next, we developed a TMEscore signature using TMEscore-related genes that were divided into TME-H and TME-L signatures. We then determined the prognostic value and association of the TMEscore signature with tumor immune scores, tumor purity, immunotherapy response, and genomic mutations, which exhibit high prognostic value and are closely associated with the above indicators. Moreover, we screened genes that represented the TMEscore signature using two machine learning algorithms and validated the predictive value of the genes using independent cohorts. Overall, the comprehensive estimation of the cellular, molecular, and genetic factors associated with TME characterization has shed light on the mechanism of tumor response to immunotherapy and may guide the identification of patients who are suitable for immunotherapy.

Consistent with previous studies regarding other types of cancers, such as gastric and prostate cancer [10, 12], patients with CRC with a high TMEscore showed a better prognosis than those with a low TMEscore. We noted that the DEGs between the TMEscore clusters were involved in immune cell processes, such as T cell activation and differentiation, and that the TMEscore clusters were related to the immune cell score and tumor purity. High TMEscores indicated more immune cells and fewer tumor cells, indicating that the TMEscore clusters were critical biomarkers for immune cells and may serve as a predictor of immunotherapy response. Currently, anti-tumor immunity through immune checkpoint inhibitors, specifically anti PD-1/PD-L1 interaction, is a new line of treatment for patients with CRC, especially for those at later stages of cancer [27]. However, only a minority of patients exhibit response to the immune checkpoint blockade, and studies have found that PD-1/PD-L1 expression and mutation load are not efficient biomarkers for predicting the benefits of immune checkpoint blockade [28, 29]. Therefore, the establishment of reliable predictive biomarkers for checkpoint immunotherapy is important for maximizing therapeutic benefits [30, 31]. In the present study, the top 10 mutated genes (*APC, TP53, KRAS, PIK3CA, ATM, PTEN, TTN, SYNE1, MUC16* and *SMAD4*), which have well documented in the pathogenesis of CRC [32–37]. Regarding the ten genes with a significant differential mutation frequency between the two signatures, mutation of EP400[38], RNF43[39], TRPM3[40],TEP1[41], MCF2[42] have been reported to associated with pathogenesis or prognosis of CRC, and the expression of DSCAML1[43], RYR1[44] and DNAH5[45] was related to CRC development. However, although ITPR3[46] and GRIK2[47] were reported to associated with cancers development, but the no evidence reported their roles in CRC. These results revealed that these TMEscore signatures also exhibited high gene mutation, and thus potentially influence the tumor microenvironment. Moreover, some genes mutation was not reported in CRC, suggesting further study is warrant to explore their role in CRC. Furthermore, we observed that the TMEscore clusters were associated with the expression of six immune checkpoint inhibitors and that genomic mutation and TMB were greatly increased in the TME-H cluster, demonstrating that patients with high TMEscores may benefit from immunotherapy.

Compared with previous studies that investigated the value of TMEscore in other cancers [10, 12, 25], our study constructed a four-gene TME signature using TMEscore-related genes (*CXCL10*, *LZTS2*, *IDO1*, and *MAB21L2*), making it easy for clinicians to assess patients who might belong to the TME-H or TME-L signature. The role of *CXCL10* and *IDO1* in CRC has been described in previous studies; for example, *CXCL10* was found to be related to immune infiltration [48], and *IDO1*(+) Paneth cells promote the immune escape of CRC [49]. LZTS2 protein reduces the level of nuclear β-catenin in CRC cells (SW480 cells) [50], while MAB21L2 is reduced in CRC and is associated with the Wnt pathway [51]. Therefore, our results suggest that further studies are required to explore their roles in that aspect. However, their roles in predicting immunotherapy have not yet been reported. Notably, *LZTS2* expression trend from GSE20916 and GSE3629 datasets was contrary to those from the other two datasets and our clinical samples. We speculated that the disparity is due to sample sources, so that the tissue stage, gene mutation status, and detection methods could influence gene expression. Hence, more studies are warranted to validate the gene expression by considering other factors related to gene expression.

In the present study, we first combined the TCGA dataset and other GEO datasets to construct a larger cohort, which could increase the reliable of the results due to the larger samples. In addition, we also employed other public datasets and clinical samples to validate the results, this strategy could guarantee the robustness of the results. Our study showed that the TMEscore is a reliable prognostic biomarker and predictive indicator in patients with CRC, and the gene expression results were verified using independent cohorts and clinical samples. Nevertheless, our results still require further validation in a prospective cohort of patients with CRC undergoing immunotherapy, and their gene expression profiles should be tested to confirm the results of the TMEscore signature. In addition, the number of immune cells in the tumors was determined by computational algorithms, the exact number of which remained to be determined using flow cytometry or immunohistochemistry. Furthermore, the treatment response of CRC is affected by numerous factors, and our study only included a few, and more clinical factors should be incorporated into predictive models to further improve the accuracy of the signature.

## Conclusion

The present study provides a comprehensive description of TME characteristics in CRC. It also demonstrates that the TME score is a potential reliable prognostic biomarker and predictive indicator for patients with CRC undergoing immunotherapy. Furthermore, our results provide a novel strategy for the precise treatment of patients with CRC.

## Data Availability

The data used to support the findings of this study are available from GEO databases(www.ncbi.nlm.nih.gov/geo/), with the access number as GSE103479, GSE29621, GSE72970, GSE39582, GSE41258, GSE20916, GSE21815, GSE3629, and GSE89287, respectively. The TCGA-COADREAD datasets was downloaded from Xena database (https://xenabrowser.net/datapages/). All data used in this study is available from the corresponding author on reasonable request.
